# Supplementary material for: A clinically applicable connectivity signature for glioblastoma includes the tumor network driver CHI3L1
Source: Nat Commun. 2024 Feb 6;15:968. doi: 10.1038/s41467-024-45067-8 (PMC10847113; doi:10.1038/s41467-024-45067-8)
Supplement: Supplementary file 1 — Supplementary Information [file 41467_2024_45067_MOESM1_ESM.pdf]

A clinically applicable connectivity signature for glioblastoma includes the tumor network driver *CHI3L1* – Supplementary information

Ling Hai[1,2,3,4]*, Dirk C Hoffmann[2,3,4]*, Robin J Wagener[2,3], Daniel D Azorin[2], David Hausmann[2,3], Ruifan Xie[2], Magnus-Carsten Huppertz[5], Julien Hiblot[5], Philipp Sievers[6,7], Sophie Heuer[2,3], Jakob Ito[2], Gina Cebulla[2], Alexandros Kourtesakis[2,3,4], Leon D Kaulen[2,3], Miriam Ratliff[2,8], Henriette Mandelbaum[2], Erik Jung[2,3], Ammar Jabali[9,10,11,12], Sandra Horschitz[9,10,11], Kati J Ernst[13,14], Denise Reibold[2], Uwe Warnken[2], Varun Venkataramani[2,3,15], Rainer Will[16], Mario L Suvà[17], Christel Herold-Mende[18], Felix Sahm[6,7], Frank Winkler[2,3], Matthias Schlesner[1,19], Wolfgang Wick[2,3], and Tobias Kessler[2,3#]

[1]Bioinformatics and Omics Data Analytics, German Cancer Research Center (DKFZ), Heidelberg, Germany
[2]Clinical Cooperation Unit Neurooncology, German Cancer Consortium (DKTK), German Cancer Research Center (DKFZ), Heidelberg, Germany
[3]Department of Neurology and Neurooncology Program, National Center for Tumor Diseases, Heidelberg University Hospital, Heidelberg, Germany
[4]Faculty of Biosciences, Heidelberg University, Heidelberg, Germany
[5]Department of Chemical Biology, Max Planck Institute for Medical Research, Heidelberg, Germany
[6]Department of Neuropathology, Institute of Pathology, University Hospital Heidelberg, Heidelberg, Germany
[7]Clinical Cooperation Unit Neuropathology, DKTK, DKFZ, Heidelberg, Germany
[8] Neurosurgery Clinic, University Hospital Mannheim, Mannheim, Germany
[9]Medical Faculty Mannheim, Central Institute of Mental Health, Heidelberg University, Mannheim, Germany.
[10]Hector Institute for Translational Brain Research, Mannheim, Germany.
[11]German Cancer Research Center (DKFZ), Heidelberg, Germany
[12]Institute of Reconstructive Neurobiology, School of Medicine & University Hospital Bonn, University of Bonn, Bonn, Germany
[13]Pediatric Glioma Research Group, DKTK, DKFZ, Heidelberg, Germany
[14]Hopp Children's Cancer Center at the NCT Heidelberg (KiTZ), Heidelberg, Germany
[15]Department of Neuroanatomy, Institute for Anatomy and Cell Biology, Heidelberg University, Heidelberg, Germany
[16]Genomics and Proteomics Core Facility, DKTK, DKFZ, Heidelberg, Germany
[17]Broad Institute of Harvard and MIT, Cambridge, MA 02142, USA; Department of Pathology and Center for Cancer Research, Massachusetts General Hospital and Harvard Medical School, Boston, MA 02114, USA
[18]Department of Neurosurgery, Heidelberg University Hospital, Heidelberg, Germany
[19]Biomedical Informatics, Data Mining and Data Analytics, Faculty of Applied Computer Science and Medical Faculty, University of Augsburg, Augsburg, Germany

* These authors contributed equally: Ling Hai, Dirk C Hoffmann
# Corresponding author: Tobias Kessler MD, Neurology Clinic and Neurooncology Program at the National Center for Tumor Diseases & DKTK, DKFZ, Im Neuenheimer Feld 400, D-69120 Heidelberg, Germany. Phone: +49 6221 56 7075, Fax: +49 6221 56 7554, E-mail: t.kessler@dkfz.de

Supplementary Figures

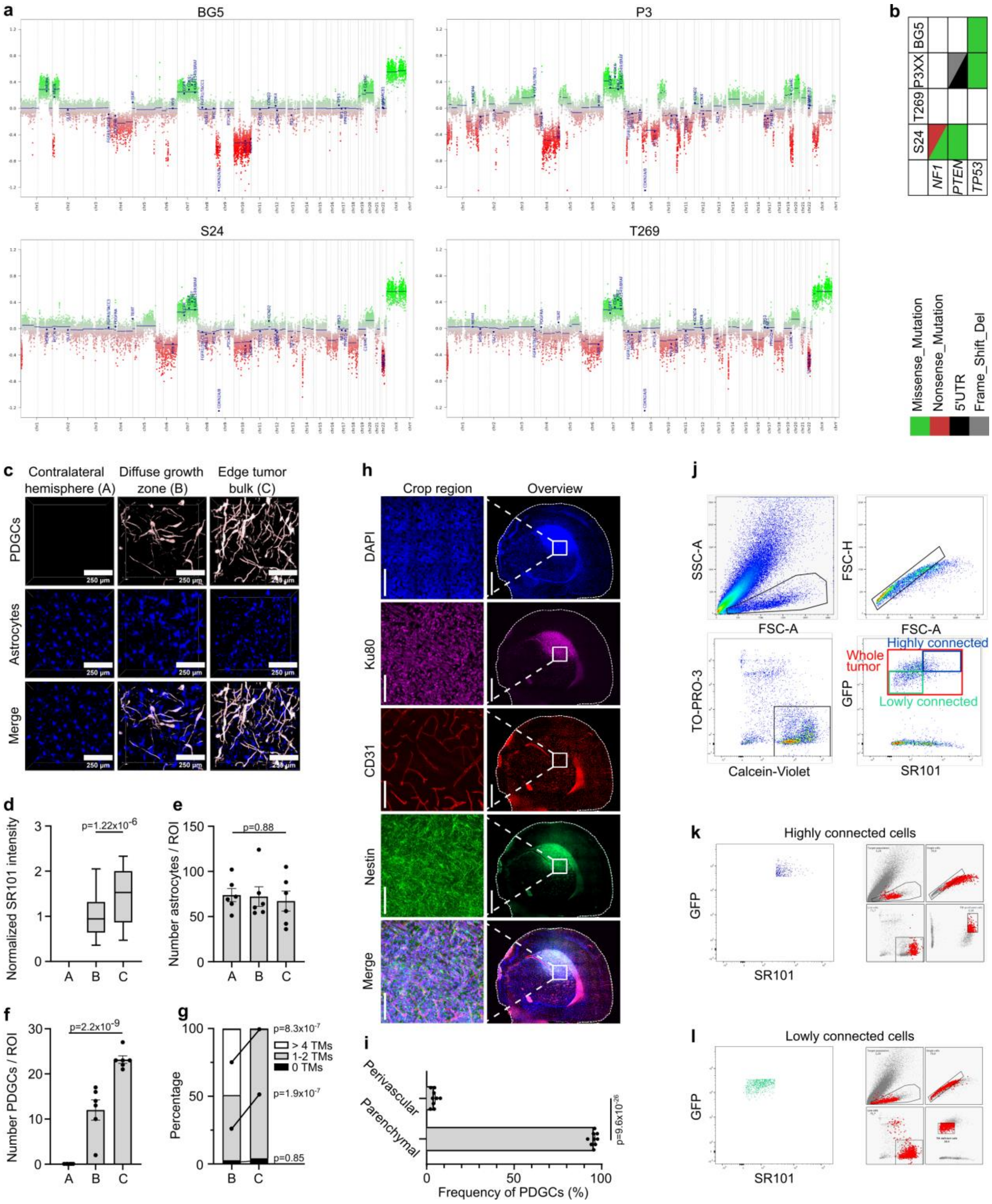

Supplementary Fig. 1: Molecular fingerprints of PDGCLs and phenotypes after orthotopic xenografting into mice. **a**, Methylation profiles of PDGCLs, images representative of n = 2 independent experiments. **b**, Genetic alterations of connectivity-signature associated genes (related to Fig. 5c). **c**, 3D segmentation of representative intravital two-photon images of TM-

connected and unconnected glioblastoma cells (grey) and astrocytes (blue) in different tumor regions, images representative of n = 3 mice. Scale bar depicts 250 µm. **d**, SR101 fluorescence intensities of tumor subpopulations located in distinct GB areas. Boxes show 25th to 75th percentile, its middle line the median, whiskers the 5th to 95th percentile and individually plotted data points the outliers. n = 69 (B) and 112 (C) PDGCs from n = 9 independent ROIs from n = 3 mice. Two-tailed unpaired t-test. **e**-**f**, Cell distribution in different GB areas. One-way ANOVA. **e**, Astrocytes. **f**, S24 PDGCs. n = 211 PDGCs and 1279 astrocytes from n = 18 regions from n = 3 mice. **g**, Number of PDGCs with distinct numbers of TMs. n = 211 PDGCs and 1,279 astrocytes from n = 18 regions from n = 3 mice. Fisher´s exact test. **h**,**i**, Blood vessel associated PDGCs are a minor subpopulation of PDGCs in vivo. **h**, Immunofluorescence micrograph of xenografted S24 PDGCs with anti-Ku-80 (magenta, nuclear marker), anti-CD31 (red, vascular marker) and anti-nestin (green, TM marker) stainings, images representative of n = 3 mice. Scale bars depict 1000 µm (overview) and 100 µm (crop region) **i**, Frequencies of PDGCs depending on their spatial association. n = 1,329 cells of n = 9 ROIs in n = 3 mice. Two-sided Mann-Whitney U test. **j**-**l**, Gating strategy to allow differentiation of tumor cell subpopulations enriched for TM-proficient (tGFP^{high}, SR101^{high}) and TM-deficient (tGFP^{high}, SR101^{low}) tumor cells, images representative of n = 3 mice. **j**, Representative analysis of three independent PDGCLs (P3, T269, S24). **k**,**l**, Backgating of TM-proficient and TM-deficient cells, respectively. **e**,**f**,**i**, Shown are the means and standard errors of the means (SEM, error bars). Exact p-values are shown in the figure. Source data are provided as a Source Data file.

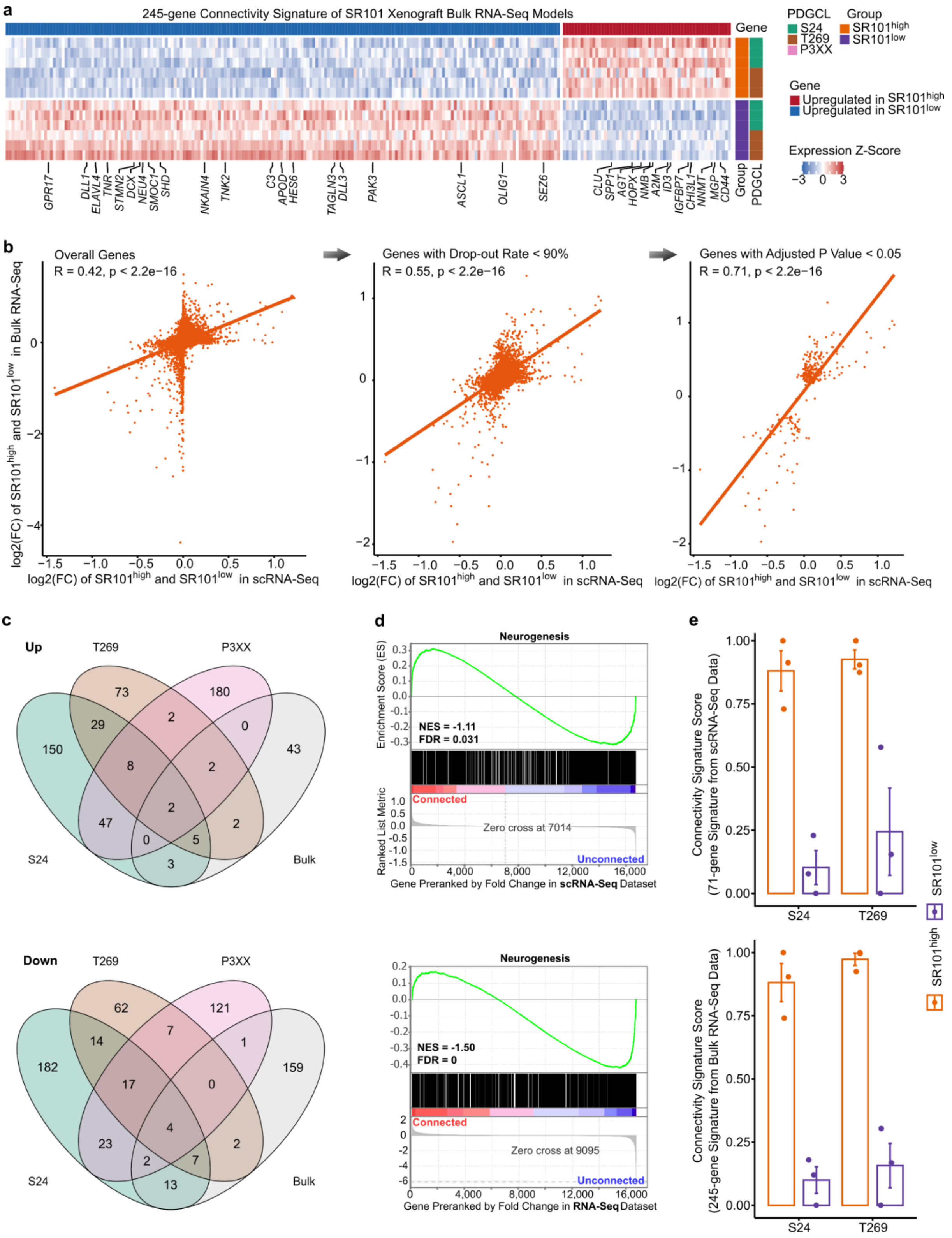

Supplementary Fig. 2: Generation and comparison of RNA-Seq and scRNA-Seq SR101 xenograft datasets. **a**, Heatmap showing expression levels of 245 RNA-Seq-derived connectivity genes in SR101^{high} and SR101^{low} samples from two xenografted patient-derived

glioblastoma cell lines (PDGCLs). Expression levels were normalized and batch effect removed. n = 3 replicates. **b,c**, Correlation of scRNA-Seq and RNA-Seq datasets of SR101 xenograft models. **b,** Scatter plots showing gene expression fold changes [log2(FC)] between $SR101^{high}$ and $SR101^{low}$ samples. Left, all detected genes (n = 16,759); Middle, genes expressed in more than 10% cells of the scRNA-Seq dataset (n = 6,984 genes); Right, genes significantly regulated between $SR101^{high}$ and $SR101^{low}$ samples in both datasets (n = 297 genes, adj. p-value < 0.05). Two-sided Spearman correlation test. **c**, Venn diagrams showing upregulated (top) and downregulated (bottom) overlapping DEGs of the bulk RNA-Seq 245-gene signature with DEGs in each PDGCL as part of the SR101 xenograft scRNA-Seq dataset. **d**, Gene set enrichment analysis for the neurogenesis gene set. Left, scRNA-Seq data. Right, RNA-Seq data. n = 16,759 genes pre-ranked by fold changes. NES: normalized enrichment score. FDR: false discovery rate. **e**, Connectivity signature score normalized to $SR101^{high}$ population in RNA-Seq dataset. Left, Score based on 71-gene connectivity signature derived from scRNA-Seq. Right, Score based on 245-gene connectivity signature derived from RNA-Seq. n = 3 replicates per group and PDGCL. Shown are the means and standard errors of the means (SEM, error bars). **a**, Values were Z-score scaled and centered across samples/PDGCs and winsorized to -3 and 3. Exact p-values are shown in the figure. Source data are provided as a Source Data file.

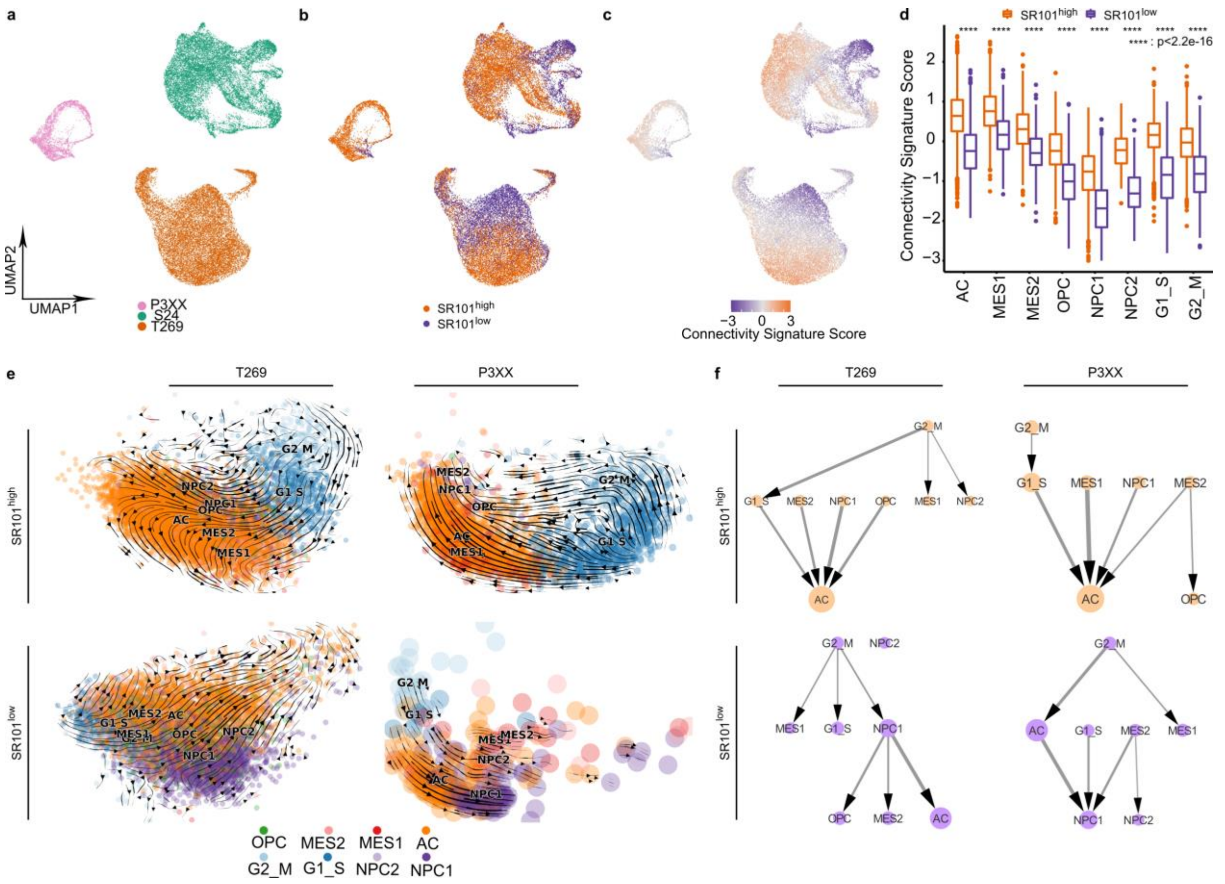

Supplementary Fig. 3: UMAPs and RNA velocities in SR101 xenograft scRNA-Seq dataset **a**-**c**, UMAPs of cells in three PDGCL xenograft mouse models without anchoring integration. **a**, Colored by the derived PDGCL. **b**, Colored by SR101 sorting. **c,** colored by connectivity signature scores. **d**, Connectivity signature score of SR101 sorting groups in each cell state. Boxes show 25th to 75th percentile, its middle line the median, whiskers the 5th to 95th percentile and individually plotted data points the outliers. n = 12,955 (AC, SR101^{high}) vs n = 3,542 (AC, SR101^{low}), n = 4,234 (MES1, SR101^{high}) vs 348 (MES1, SR101^{low}), n = 1,038 (MES2, SR101^{high}) vs n = 689 (MES2, SR101^{low}), n = 421 (OPC, SR101^{high}) vs n = 873 (OPC, SR101^{low}), n = 1,370 (NPC1, SR101^{high}) vs n = 4,212 (NPC1, SR101^{low}), n = 127 (NPC2, SR101^{high}) vs n = 195 (NPC2, SR101^{low}), n = 2,587 (G1_S, SR101^{high}) vs n = 936 (G1_S, SR101^{low}), n = 1,428 (G2_M, SR101^{low}), n = 867 (G2_M, SR101^{high}) vs n = 936 (G2_M, SR101^{low}) PDGCs from n = 3 mice per group. Two-sided Mann-Whitney U test. **e**, RNA velocities projected on PCA embedding of T269 and P3XX PDGCs. Streamline indicates the directional flow. Each dot is

one single PDGC colored by cell state. **f**, Directed PAGA graphs based on RNA velocity analysis in **e**. Each dot is one cell state and the size of dot indicates the number of cells in the cell state. The width of the arrow indicates the transition possibility between cell states. **c**,**d**, Scores were scaled, centered and winsorized at -3 and 3 across cells. Exact p-values are shown in the figure. Source data are provided as a Source Data file.

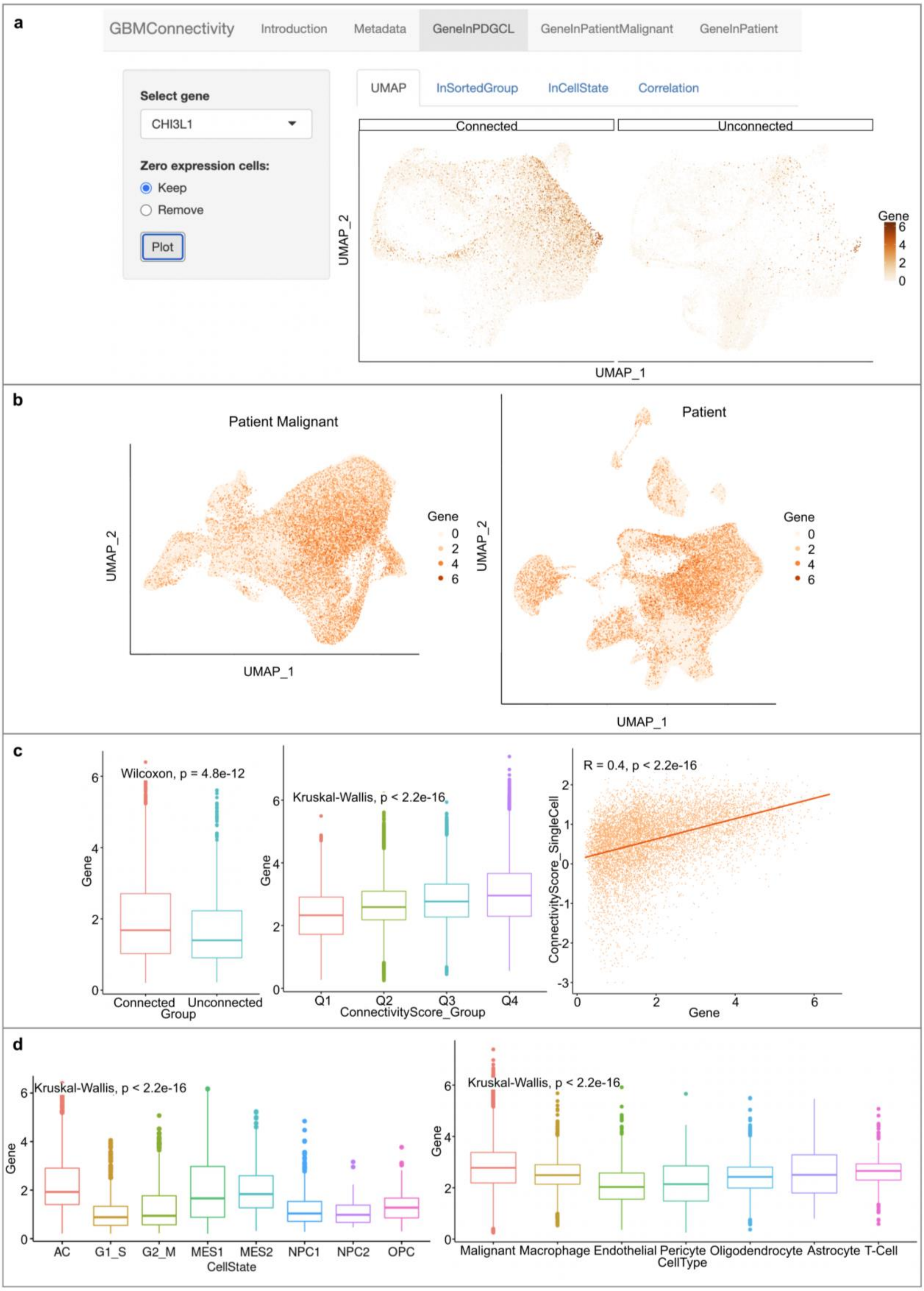

Supplementary Fig. 4: Online webtool for visualization of SR101 xenograft scRNA-Seq and GB patient snRNA-Seq datasets with analysis of user-selected genes (https://conectivity-

glioma.dkfz.de). Data include 35K cells from PDGCL xenograft mouse models and 213K cells (172K malignant and 41K nonmalignant cells) from 21 GBM patient samples. Meta data contains information of samples, SR101-sorted groups, cell states, cell types, and connectivity signature scores derived from the scRNA-Seq and RNA-Seq datasets. Meta data of cells are visualized in UMAPs (in the MetaData panel). User can analyze genes of interest interactively, for example: showing UMAPs of normalized expression levels of a user-selected gene in highly connected and lowly connected cells from the PDGCL dataset (**a**), or in patient malignant cells (**b**, Left), or in all cells from patient samples (**b**, Right); showing box plots of gene expression levels in highly connected and lowly connected cells from the PDGCL dataset (**c,** Left), or in connectivity signature score groups (grouped by quantiles) from patient malignant cells (**c,** Middle); showing scatter plot of gene expression levels and connectivity signature scores in the PDGCL dataset (**c**, Right); showing box plot of gene expression levels in cell states in the PDGCL dataset (**d**, Left), or in cell types in the patient sample dataset (**d**, Right).

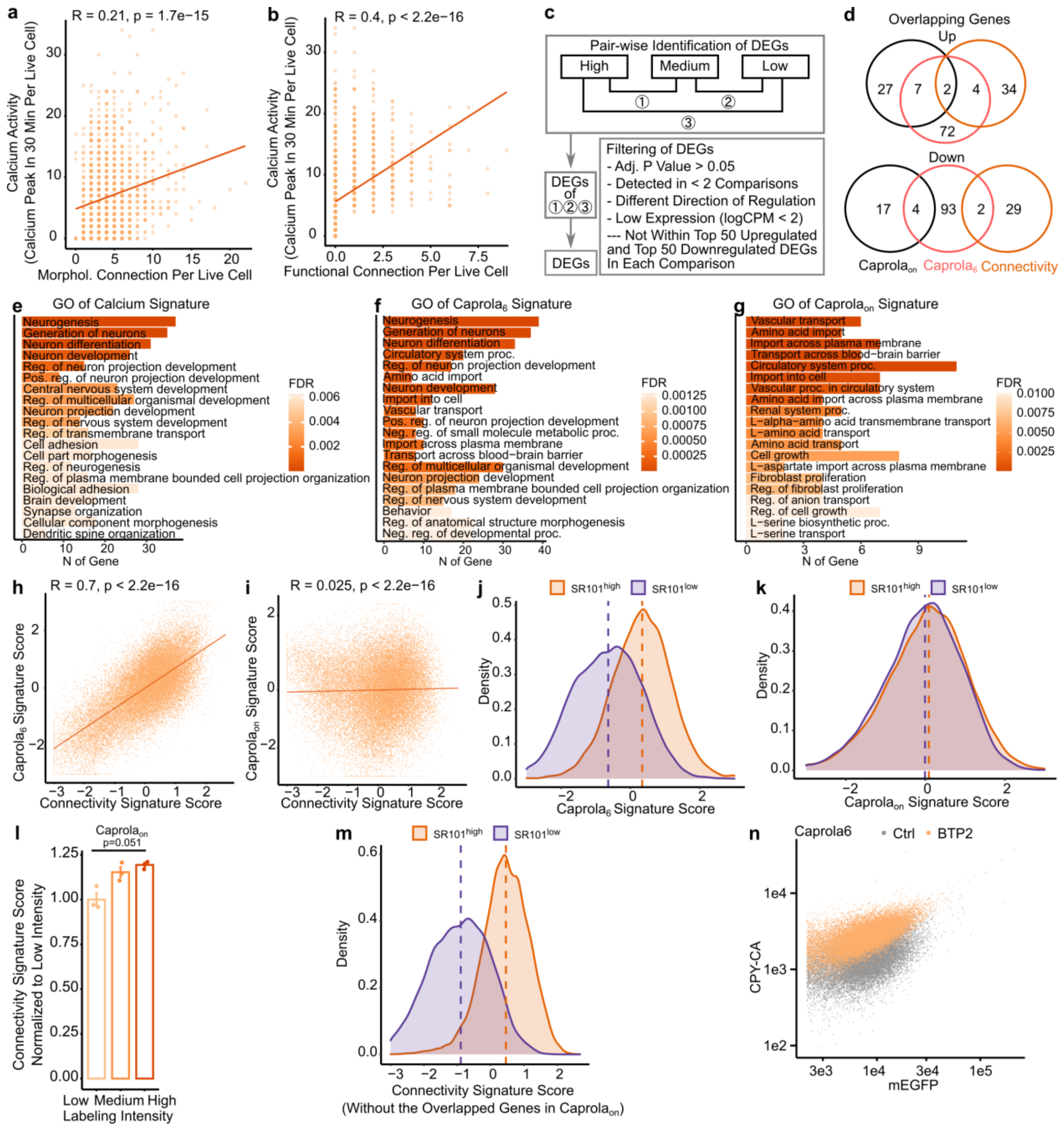

Supplementary Fig. 5: TM networks are characterized by Ca^{2+} activity and can be modeled in vitro. **a**,**b**, Correlation of morphological and functional connections with Ca^{2+} activity per S24 live PDGC. n = 1,357 PDGCs from n = 3 recordings. Two-sided Spearman correlation test. **a**, Morphological connections versus Ca^{2+} activity. **b**, Functional connections versus Ca^{2+} activity. **c-n**, Comparison of Ca^{2+}-dependent and Ca^{2+}-independent Caprola-models used for the characterization of PDGCs with differential calcium activity. **c**, Bioinformatic generation of the Caprola$_6$ and Caprola$_{on}$ signatures (see methods). **d**, Venn diagram showing the number of

overlapping upregulated and downregulated genes of the $Caprola_6$ signature, $Caprola_{on}$ signature and Connectivity signature. **e-g**, Enriched GOs of signatures. Top 20 GOs were ordered by false discovery rate (FDR). **e**, 171-gene calcium signature. **f**, 184-gene $Caprola_6$ signature. **g**, 57-gene $CaProLa_{on}$ signature. **h**,**i**, Scatter plot showing correlation of two signature scores in SR101 xenograft scRNA-Seq dataset. n = 35,822 PDGCs. Two-sided Pearson correlation test. **h**, $Caprola_6$ signature score versus connectivity signature score. **i**, $Caprola_{on}$ signature score versus connectivity signature score. **j**,**k**, Density plot of signature scores in $SR101^{high}$ and $SR101^{low}$ populations. Dotted lines depict median. **j**, $Caprola_6$ signature score. **k**, $Caprola_{on}$ signature score. **l**, Connectivity signature scores in S24-$Caprola_{on}$ populations with high, medium and low labeling intensity ratios. Scores were normalized to labeling $intensity^{low}$ population. Shown is the mean and standard error of the mean (SEM, error bars). n = 3 replicates per group. Two-sided Kruskal-Wallis test. **m**, Density plot of connectivity signature scores (without the overlapped genes in $Caprola_{on}$ signature) in $SR101^{high}$ and $SR101^{low}$ populations. Dotted lines depict median. **n**, FACS analysis of S24-$Caprola_6$ PDGCs after Ctrl or BTP2 treatment. n = 21,792 PDGCs (Ctrl) vs n = 20,662 PDGCs (BTP2) of n = 2 replicates. Exact p-values are shown in the figure. Source data are provided as a Source Data file.

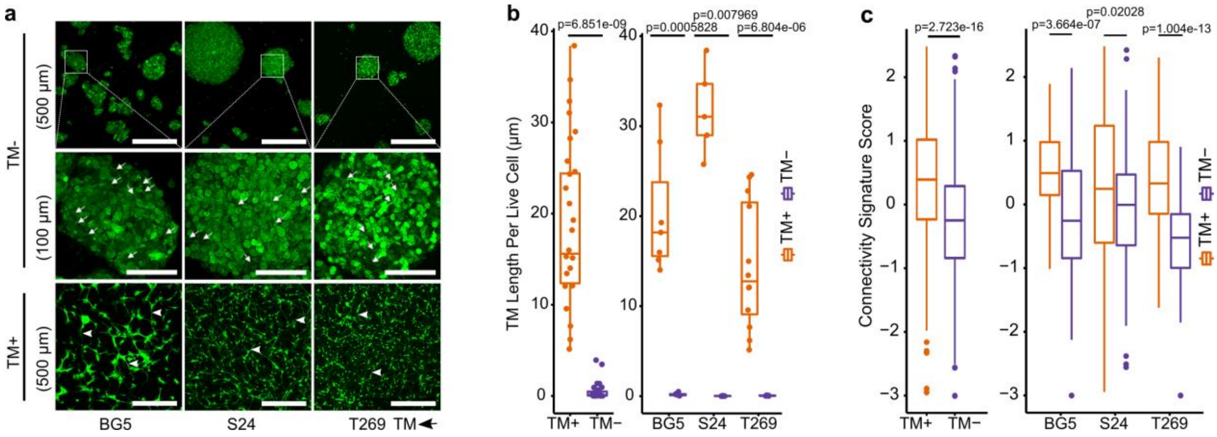

Supplementary Fig. 6: A serum-containing in vitro model of morphological GB networks. **a**, Fluorescence micrographs from three PDGCLs, images representative of n = 2 independent experiments. Top, cells cultured as neurospheres, TM-. Scale bars depict 500 µm. Middle, zoom in from the top panel. Scale bars depict 100 µm. Arrows indicate TMs. Bottom, PDGCs cultured in serum-containing media, TM+. Scale bars depict 500 µm. **b**, TM lengths per live cell in n = 7 ROIs (BG5, TM+) vs n = 7 ROIs (BG5, TM-), n = 5 (S24, TM+) vs n = 6 (S24, TM-), n = 12 (T269, TM+) vs n = 9 (T269, TM-) with similar nuclei density of n = 2 independent experiments. Left, PDGCs from all three PDGCLs. Right, separated in each PDGCL. Two-sided t-test. **c**, Connectivity signature scores in single cells. Left, cells from all three PDGCLs. Right, separated in each PDGCL. Connectivity signature scores were Z-score scaled and centered across cells and winsorized to -3 and 3. n = 71 PDGCs (BG5, TM+) vs n = 92 (BG5, TM-), n = 85 (S24, TM+) vs n = 135 (S24, TM-), n = 93 (T269, TM+) vs n = 90 PDGCs (T269, TM-). Two-sided t-test. **b**,**c**, Boxes show 25th to 75th percentile, its middle line the median, whiskers the 5th to 95th percentile and individually plotted data points the outliers. Exact p-values are shown in the figure. Source data are provided as a Source Data file.

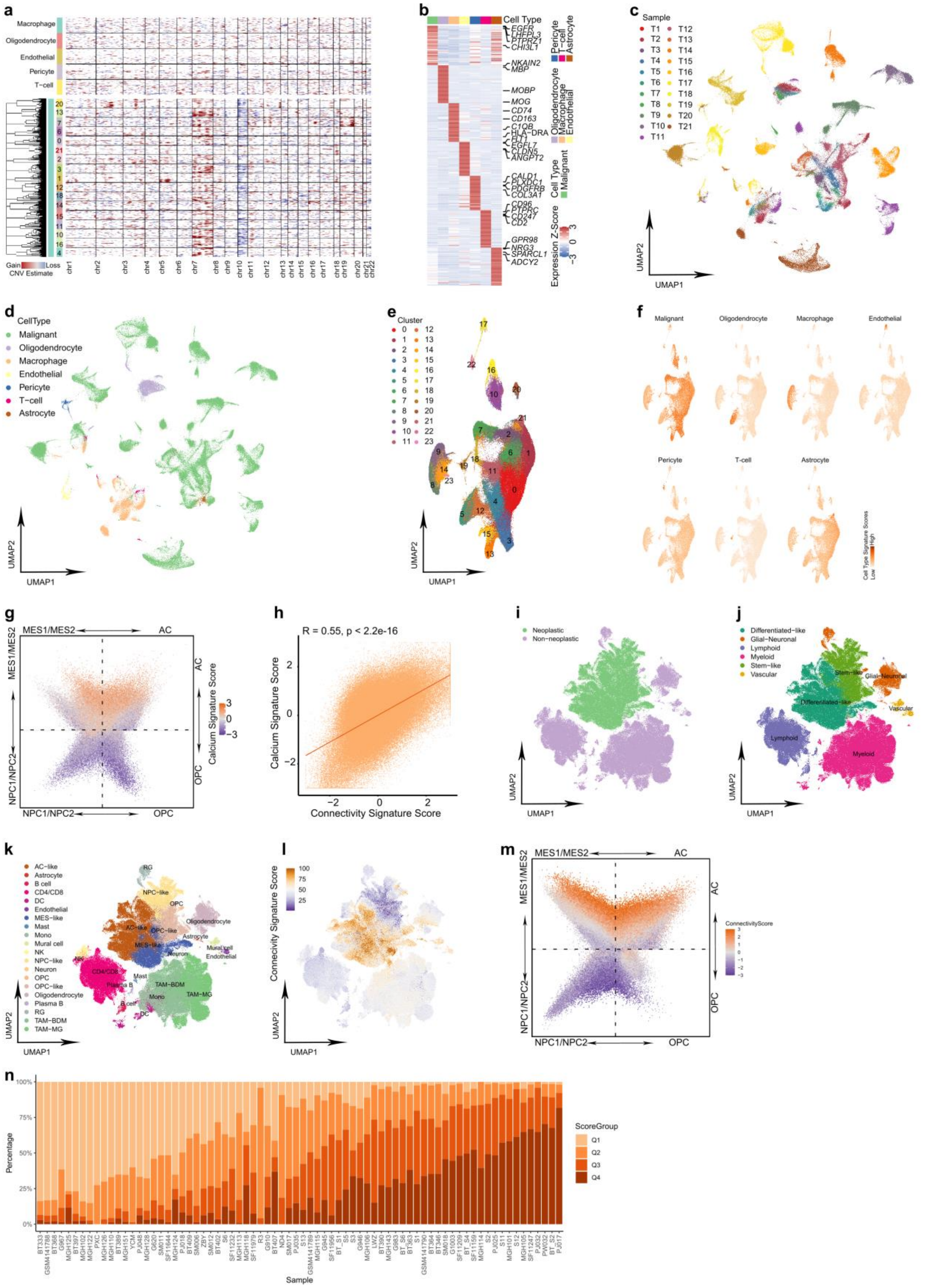

Supplementary Fig. 7: snRNA-seq of GB patient samples. **a-g**, snRNA-seq of n = 21 GBM patient samples (n = 213,444 cells). **a**, CNVs in non-malignant cell types and malignant clusters.

Cluster 21 marked in red indicates the non-malignant astrocyte cluster. **b**, Heatmap of average expression levels of top 50 markers per cell type. Expression levels were scaled and centered across cell types and sinsorized to -3 and 3. **c-d**, UMAPs of cells from 21 patient samples without anchoring integration. **c**, colored by patient. **d**, Colored by malignant and non-malignant cell types. **e-f**, UMAPs of patient samples with anchoring integration. **e**, Colored by unsupervised clusters. 24 clusters were obtained. **f**, Cell type signature scores of malignant and each non-malignant cell types. **g**, Two-dimensional representation of patient malignant cells according to cell state signature scores. Cells were colored by calcium signature scores. **h**, Scatter plot showing correlation between calcium signature score and connectivity signature scores. Pearson correlation test. **e-g**, Scores or gene expression were Z-score scaled, centered and winsorized to -3 and 3. **i-n**, snRNA-seq dataset of n = 110 GB specimen[1] (n = 338,564 cells). **i-l**, UMAPs of patient samples with anchoring integration. **i**, Colored by malignancy. **j**, Colored by major population. **k**, Colored by subclustered cell type/state composition. **l**, Colored by connectivity signature score. **m**, Two-dimensional representation of patient malignant cells according to cell state signature scores. n = 125,486 cells from n = 74 donors. Cells were colored by connectivity signature scores. **n**, Frequency of connectivity signature score groups in malignant cells. A connectivity signature score is calculated for each cell and it is then assigned to one of the four score groups (lower score quartile [Q1] - highest score quartile [Q4]). Exact p-values are shown in the figure. Source data are provided as a Source Data file.

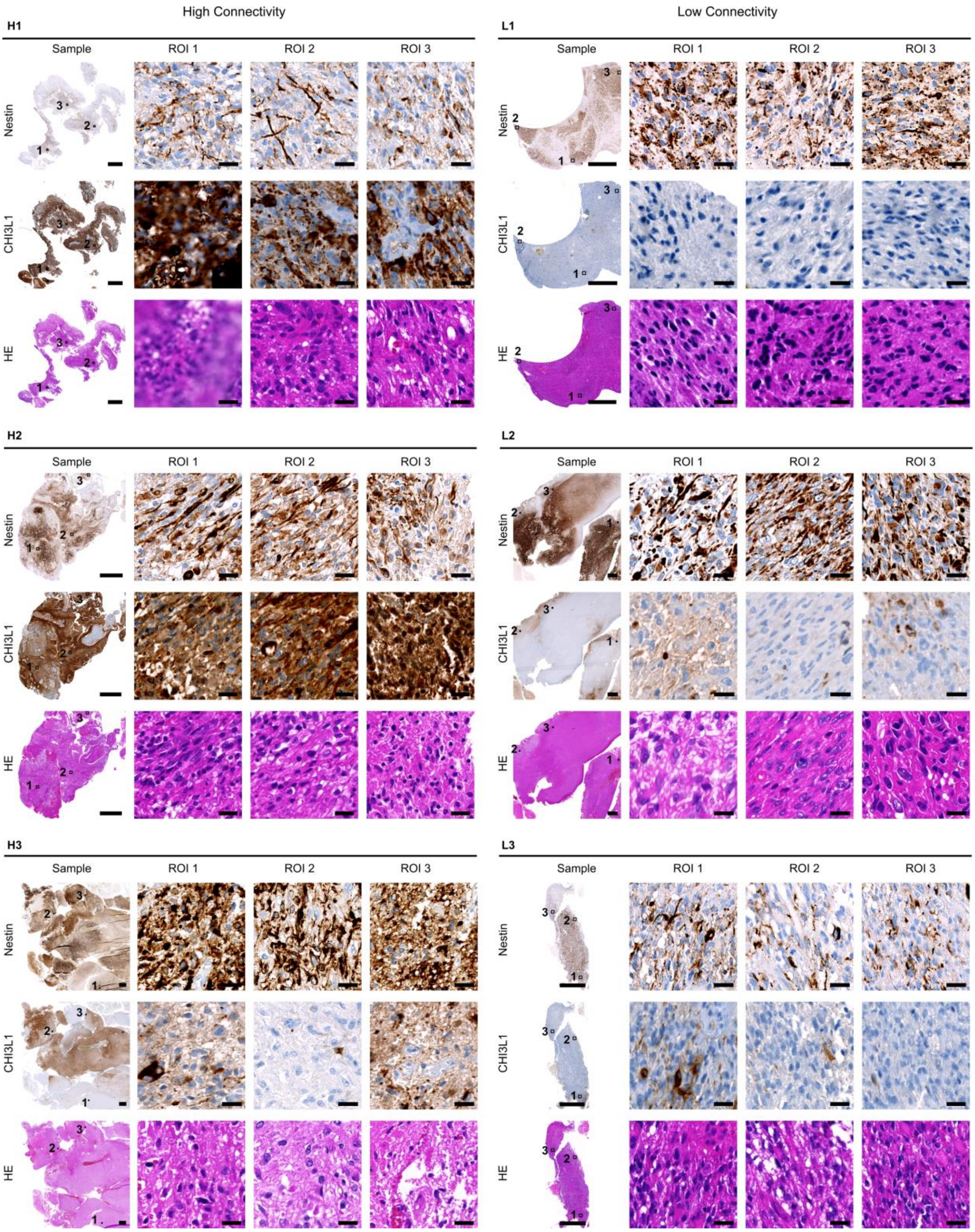

Supplementary Fig. 8: IHC micrographs with anti-nestin, anti-CHI3L1, and HE stainings of six GB patients. Left column shows three patients with high connectivity signature scores (H1, H2 and H3), right column shows three patients with low connectivity signature scores (L1, L2 and L3). In each patient, row shows staining of nestin, CHI3L1 or HE; column shows image

with different size and regions. In the Sample column, three marked regions indicate ROIs displayed in the next three columns (ROI1, ROI2 and ROI3). Scale bars of samples depict 1000 µm. Scale bars of ROIs depict 20 µm.

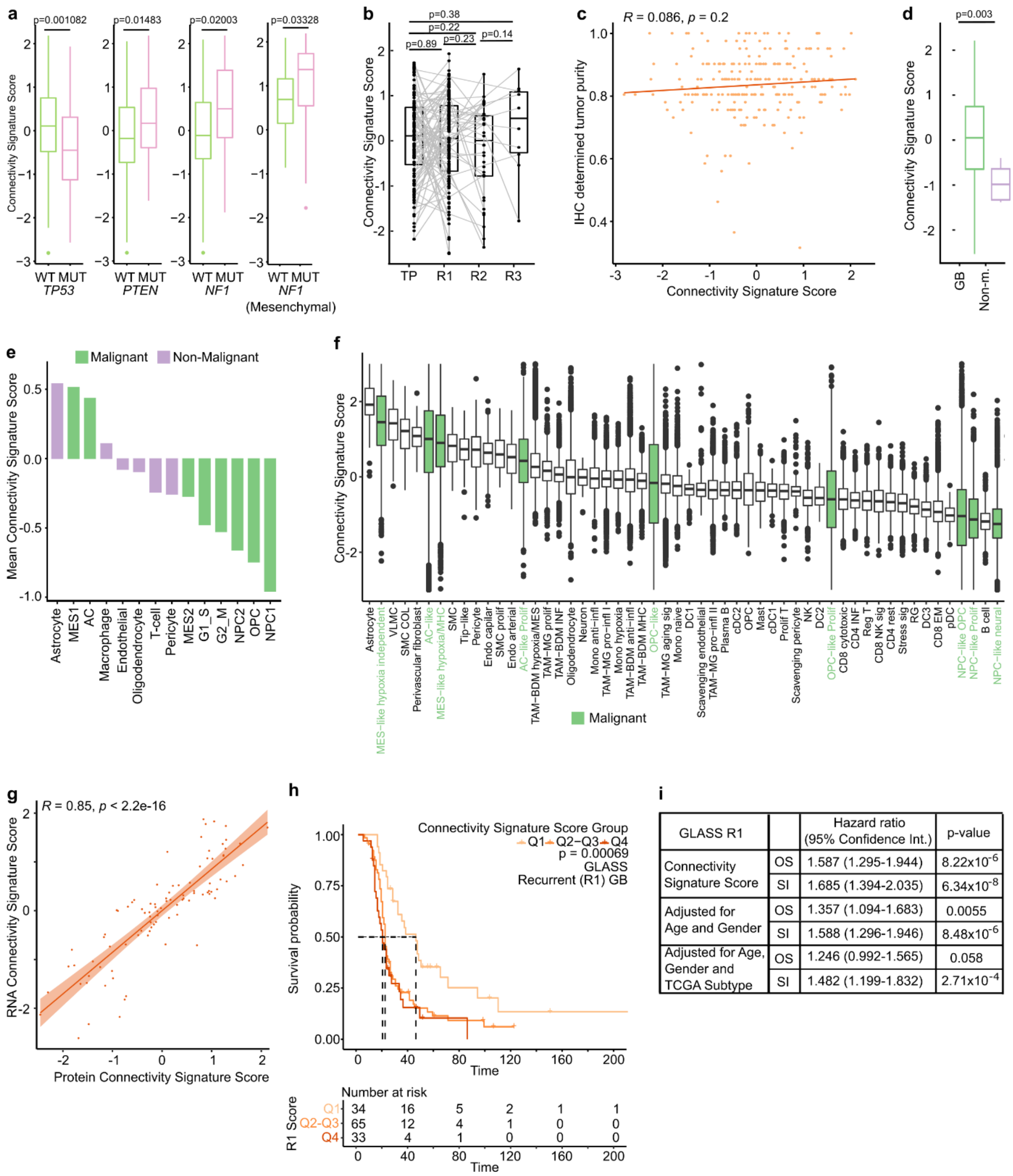

GLASS R1		Hazard ratio (95% Confidence Int.)	p-value
Connectivity Signature Score	OS	1.587 (1.295-1.944)	8.22×10^{-6}
	SI	1.685 (1.394-2.035)	6.34×10^{-8}
Adjusted for Age and Gender	OS	1.357 (1.094-1.683)	0.0055
	SI	1.588 (1.296-1.946)	8.48×10^{-6}
Adjusted for Age, Gender and TCGA Subtype	OS	1.246 (0.992-1.565)	0.058
	SI	1.482 (1.199-1.832)	2.71×10^{-4}

Supplementary Fig. 9: Features of the connectivity signature in large cohorts. **a**, Connectivity signature scores in TCGA grouped by mutation status. n = 173 (*TP53* wt) vs n = 57 (*TP53* mut), n = 154 (*PTEN* wt) vs n = 76 (*PTEN* mut); n = 195 (*NF1* wt) vs n = 35 (*NF1* mut), n = 63 (*NF1* wt) vs n = 16 (*NF1* mut) patients in MS subtype. Two-sided Mann-Whitney U test. **b**, Connectivity signature scores in primary and recurrent GB samples. n = 161 primary (TP), n = 166 first recurrent (R1), n = 34 second recurrent (R2), n = 10 third recurrent (R3) samples.

Lines link identical patients. Two-sided Mann-Whitney U test. **c**, Correlation between IHC determined tumor purity and Connectivity signature scores. n = 230 TCGA GB. Two-sided Pearson correlation test. **d-f**, Connectivity signature scores in bulk tissues and cell populations. **d**, Connectivity signature scores in RNA-Seq of TCGA GB (n = 230) and GTEx healthy brain (n = 7) samples. Two-sided Mann-Whitney U test. **e**, Connectivity signature scores in malignant and non-malignant cell types in our snRNA-Seq patient dataset (related to Fig. 5). **f**, Connectivity signature scores of cell types annotated in the GBmap dataset[1], n = 338,564 cells from n = 110 GB specimen. **g**, Correlation of connectivity signature scores between transcriptome and proteome profiles of bulk GB. n = 93 bulk GB patient specimens. Two-sided Pearson correlation test. **h**, Kaplan-Meier survival analysis of GLASS cohort patients stratified into three score groups of connectivity signature score (lower score quartile [Q1] - highest score quartile [Q4]). Connectivity signature scores were derived from recurrent (R1) GB tumors. n = 133. **i**, Cox proportional hazards regression surgical interval (SI) and overall survival (OS) analysis in the recurrent (R1) GB GLASS cohort. **a-f**, Scores or gene expression were Z-score scaled, centered and winsorized to -3 and 3. **a**,**b**,**d**,**f**, Boxes show 25th to 75th percentile, its middle line the median, whiskers the 5th to 95th percentile and individually plotted data points the outliers. Exact p-values are shown in the figure. Source data are provided as a Source Data file.

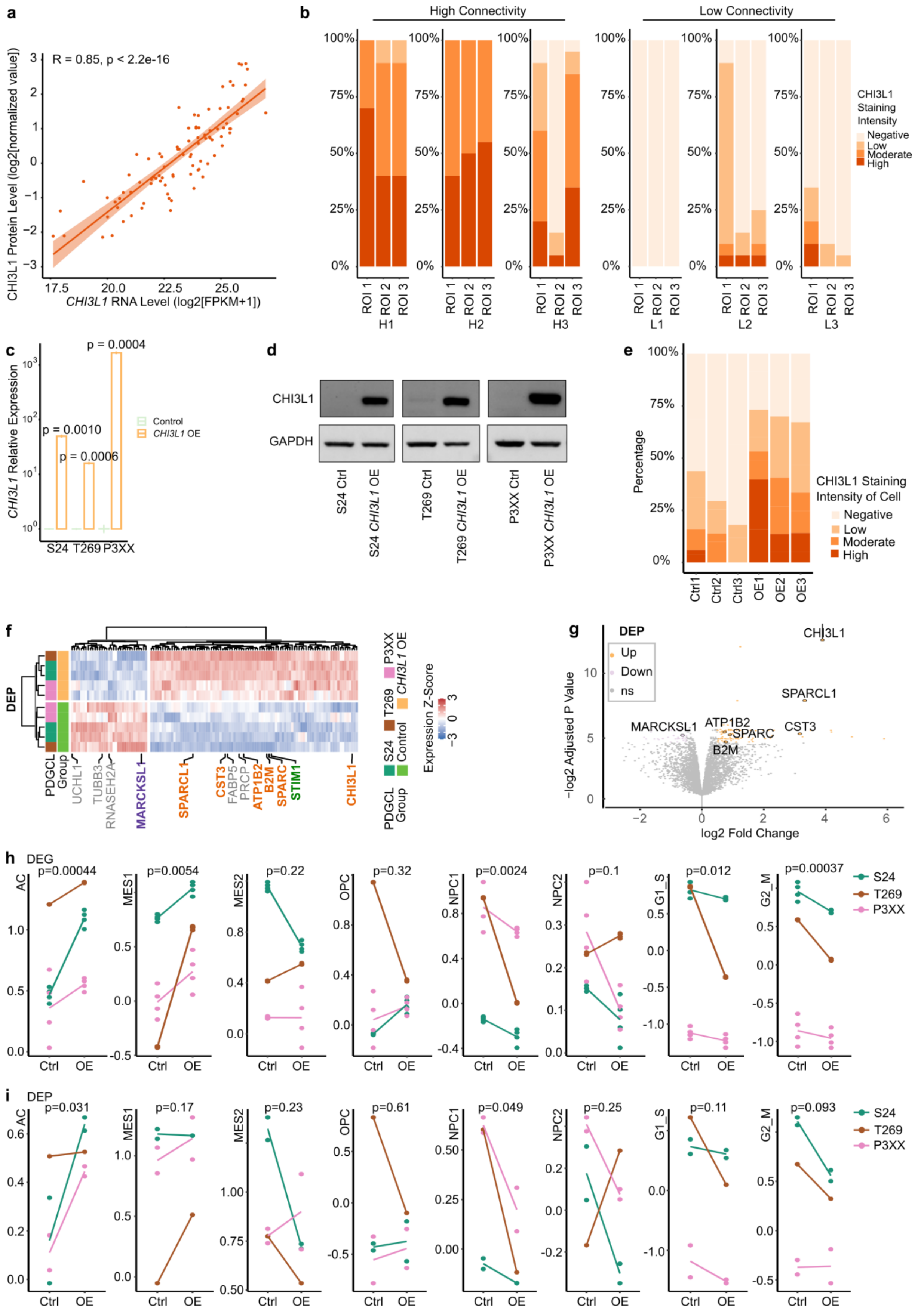
a
R = 0.85, p < 2.2e-16
CHI3L1 Protein Level (log2[normalized value])
CHI3L1 RNA Level (log2[FPKM+1])
b
High Connectivity
Low Connectivity
CHI3L1 Staining Intensity
Negative
Low
Moderate
High
H1
H2
H3
L1
L2
L3
c
CHI3L1 Relative Expression
p = 0.0004
p = 0.0010
p = 0.0006
Control
CHI3L1 OE
S24 T269 P3XX
d
CHI3L1
GAPDH
S24 Ctrl
S24 CHI3L1 OE
T269 Ctrl
T269 CHI3L1 OE
P3XX Ctrl
P3XX CHI3L1 OE
e
Percentage
CHI3L1 Staining Intensity of Cell
Negative
Low
Moderate
High
Ctrl1 Ctrl2 Ctrl3 OE1 OE2 OE3
f
DEP
PDGCL Group
UCHL1
TUBB3
RNASEH2A
MARCKSL1
SPARCL1
CST3
FABP5
PRCP
ATP1B2
B2M
SPARC
STIM1
CHI3L1
P3XX
CHI3L1 OE
T269
Control
S24
Expression Z-Score
g
DEP
Up
Down
ns
CHI3L1
SPARCL1
MARCKSL1
ATP1B2
SPARC
CST3
B2M
-log2 Adjusted P Value
log2 Fold Change
h DEG
AC p=0.00044
MES1 p=0.0054
MES2 p=0.22
OPC p=0.32
NPC1 p=0.0024
NPC2 p=0.1
G1_S p=0.012
G2_M p=0.00037
S24
T269
P3XX
i DEP
AC p=0.031
MES1 p=0.17
MES2 p=0.23
OPC p=0.61
NPC1 p=0.049
NPC2 p=0.25
G1_S p=0.11
G2_M p=0.093
Ctrl OE

Supplementary Fig. 10: *CHI3L1* is a robust marker for TM-connectivity with functional relevance. **a**, Scatter plot showing correlation between *CHI3L1* mRNA expression level (log2(FPKM+1)) and protein levels in *IDH* wt GB samples from n = 93 samples. Two-sided Pearson correlation test. **b**, Frequencies of cellular CHI3L1 staining intensity (i.e., negative, low, moderate and high) in ROIs shown in Supplementary Fig. 8. **c**, Bar plot of *CHI3L1* relative gene expression measured by qPCR in three Ctrl or *CHI3L1* overexpression (OE) PDGCLs. Shown are the means and standard error of the means (SEM, error bars). n = 6 replicates of n = 2 independent experiments. Two-sided t-test. **d**, Western blot of *CHI3L1* protein expression in three Ctrl or *CHI3L1* overexpression (OE) PDGCLs, image representative of n = 2 independent experiments. Uncropped blots are available in the Source Data file. **e**, Frequency of cellular CHI3L1 staining intensity of xenografted S24 Ctrl and *CHI3L1* OE PDGCs. **f**, Heatmap showing differentially expressed protein (DEP) intensities in Ctrl and *CHI3L1* OE PDGCLs. Data were Z-score scaled and centered across samples, and winsorized to -3 and 3. Colors: Purple, downregulated connectivity signature genes; Orange, upregulated connectivity signature genes; Gray, Neftel cell state signatures. **g**, Volcano plot comparing DEPs of Ctrl and *CHI3L1* OE PDGCLs P3XX, S24 and T269. Proteins and phosphosites shown in orange or purple have adj. p-value < 0.05. The overlapping genes with connectivity signature are depicted. P_{adj} is the adjusted value statistically corrected for multiple testing. **h**-**i**, Cell state signature scores in Ctrl and *CHI3L1* OE in PDGCLs P3XX, S24 and T269. Lines indicate the average in each PDGCL. **h**, DEGs. **i**, DEPs. Lines indicate the average in each PDGCL. n = 4 independent replicates per PDGCL in RNA-Seq. n = 2 independent replicates (S24 and T269) and n = 1 independent replicate (P3XX) in proteomics. Two-sided paired t-test. Exact p-values are shown in the figure. Source data are provided as a Source Data file.

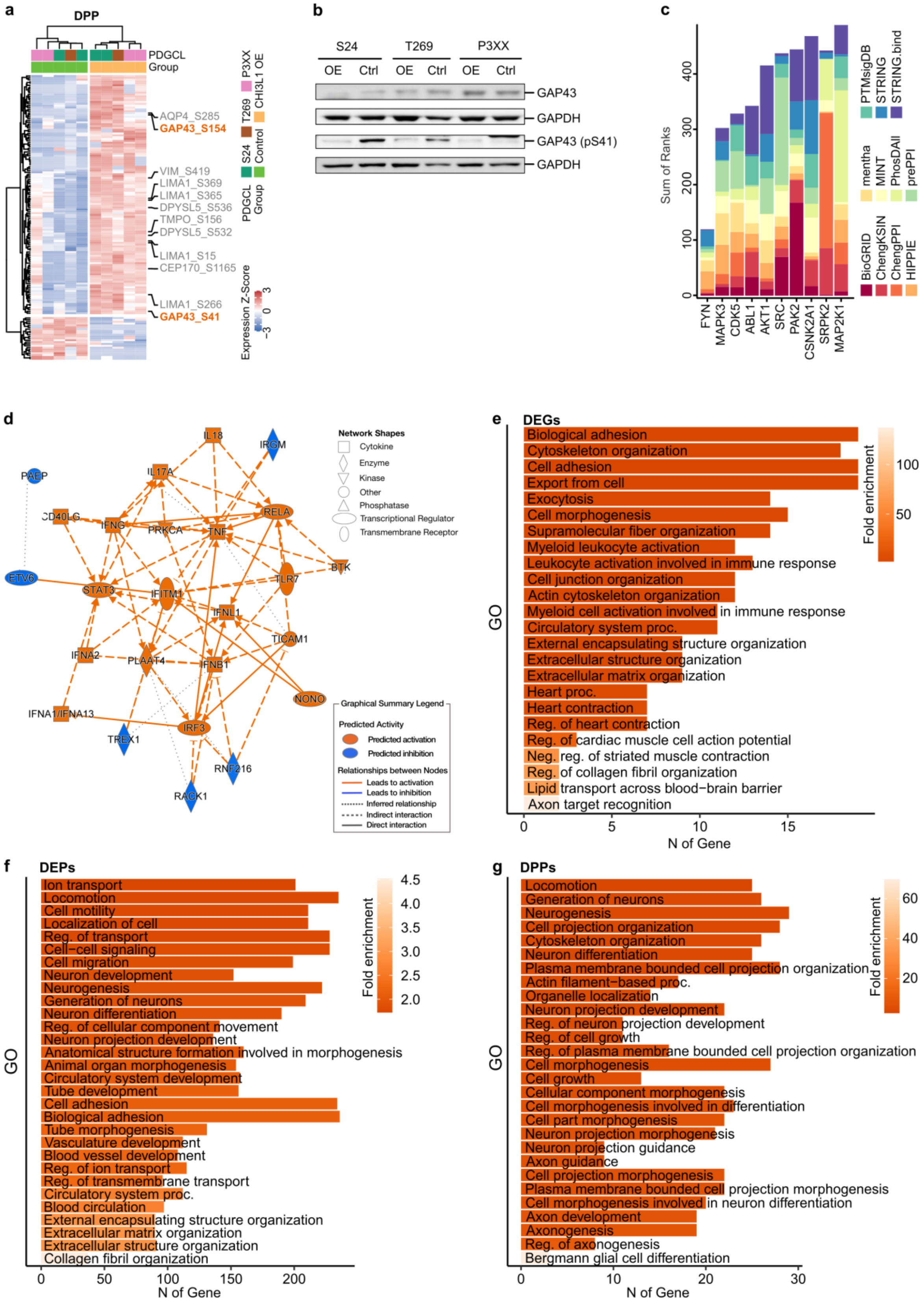

Supplementary Fig. 11: Pathway analysis of Ctrl and *CHI3L1* OE PDGCLs

a, Heatmap showing differentially expressed protein phosphosite (DPP) intensities in Ctrl and *CHI3L1* OE PDGCLs. Data were Z-score scaled and centered across samples, and winsorized to -3 and 3. Colors: Orange, Detected differentially expressed GAP43 phosphorylation sites. **b**, Western blot with GAP43 and phosphorylated S41 of GAP43, image representative of n = 2 independent experiments. Uncropped blots are available in the Source Data file. GAPDH serves as a loading control. **c**, Kinase enrichment analysis of *CHI3L1* OE PDGCs. MEK/ERK and AKT pathways were activated in *CHI3L1* OE PDGCs. **d**, Graphical summary of Ingenuity Pathway Analysis of DEGs. STAT3 was predicted to be activated and a signaling hub in *CHI3L1* OE PDGCLs. **e-g** Top 30 enriched GO terms (FDR < 0.05) ordered by enrichment fold-changes. X-axis showing the number of overlapping genes between DEGs and GO genes. Color indicates the enrichment fold-changes. **e**, GOs of DEGs. **f**, GOs of DEPs. **g**, GOs of DPPs. Exact p-values are shown in the figure. Source data are provided as a Source Data file.

Supplementary Tables

Supplementary Table 1. Properties of PDGCLs.

**Name**	**Age**	**Sex**	**IDH**	**MGMT**	**Methylation subtype**	**TCGA expression subtype**	**Dominant cell state**
**S24**	Range 43-72	F	WT	M	RTK I	MS	MES1
**T269**	Range 43-72	M	WT	M	RTK II	CL	AC
**P3XX**	Range 43-72	M	WT	M	RTK II	CL	MES2
**BG5**	Range 43-72	F	WT	U	RTK II	PN	NPC1

F, female; M, male; WT, wildtype; M, promoter methylated; U, promoter unmethylated; RTK, receptor tyrosine kinase; MS, mesenchymal; CL, classical; PN, proneural; AC, astrocyte-like; MES2, mesenchymal-like 2; NPC1, neural-progenitor-like 1

Supplementary Table 2. Properties of PDGCs part of the SR101 xenograft scRNA-Seq dataset.

**Sample**	**PDGCL**	**Group**	**Genes (n)**	**UMIs (n)**	**Cells (n)**
**S24-SR101-high**	S24	SR101-high	2876	7994	11190
**S24-SR101-low**	S24	SR101-low	3153	8710	4439
**T269-SR101-high**	T269	SR101-high	1380	2659	10245
**T269-SR101-low**	T269	SR101-low	1590	3361	6933
**P3XX-SR101-high**	P3XX	SR101-high	4718	23140	2725
**P3XX-SR101-low**	P3XX	SR101-low	5230	26020	290

Supplementary Table 3. The prediction performances of connectivity signature scores against SR101-sorted labels.

**Metrics**	**245-gene signature derived from RNA-Seq**	**71-gene connectivity signature derived from scRNA-Seq**	**245 randomly generated genes**	**71 randomly generated genes**
Accuracy	0.79	0.83	0.49	0.49
Sensitivity	0.77	0.95	0.47	0.48
Specificity	0.83	0.58	0.53	0.51
PPV	0.90	0.82	0.67	0.67
NPV	0.64	0.84	0.32	0.32

PPV, positive predictive value; NPV, negative predictive value.

Supplementary Table 4. Clinical characteristics and quality measures of snRNA-Seq patients.

ID	Age	Sex	IDH	MGMT	GBM meth. class	Primary/ recurrent	Median gene (n)	Median count (n)	Cell (n)	Malignant cell (n)
T1	Range 32-80	Male	WT	U	RTK I	primary	561	685	932	800
T2	Range 32-80	Male	WT	U	Mesenchymal	recurrent	786	1116	16721	6423
T3	Range 32-80	Male	WT	U	RTK I	primary	634	794	6634	6184
T4	Range 32-80	Male	WT	U	RTK II	primary	943	1273	9029	7455
T5	Range 32-80	Male	WT	U	RTK II	primary	767	1006	6175	3232
T6	Range 32-80	Male	WT	U	Mesenchymal	primary	1020	1440	2744	1916
T7	Range 32-80	Male	WT	U	RTK II	primary	643	846	5009	2757
T8	Range 32-80	Female	WT	U	Mesenchymal	primary	584	679	1626	1552
T9	Range 32-80	Male	WT	U	Mesenchymal	primary	1393.5	2071	15092	13737
T10	Range 32-80	Male	WT	U	Mesenchymal	primary	1097	1653.5	11192	10573
T11	Range 32-80	Male	WT	M	N/A	primary	1198	1762	14588	10918
T12	Range 32-80	Male	WT	M	RTK I	primary	996	1381	15057	13751
T13	Range 32-80	Male	WT	U	RTK I	primary	1231	1946	5165	4526
T14	Range 32-80	Female	WT	U	RTK II	primary	1289	2053	11927	10795
T15	Range 32-80	Male	WT	M	RTK II	primary	998	1310	11533	11081
T16	Range 32-80	Female	WT	U	RTK II	primary	608	728	5830	5600
T17	Range 32-80	Male	WT	U	Mesenchymal	primary	1347.5	1917	19668	16840
T18	Range 32-80	Male	WT	U	Mesenchymal	primary	810	1102	8221	5140
T19	Range 32-80	Male	WT	U	RTK I	primary	1300.5	1890	13450	10963
T20	Range 32-80	Female	WT	U	Mesenchymal	primary	856	1055	15707	15140
T21	Range 32-80	Male	WT	U	RTK II	primary	1912	3024.5	17144	13088

N, number; WT, wildtype; M, promoter methylated; RTK, Receptor tyrosine kinase; U, promoter unmethylated; censor 1, progressive disease; censor 0, stable disease.

References

1. Ruiz-Moreno, C. *et al. Harmonized single-cell landscape, intercellular crosstalk and tumor architecture of glioblastoma* (2022).
