# Supplementary material for: A clinically applicable connectivity signature for glioblastoma includes the tumor network driver CHI3L1
Source: Nat Commun. 2024 Feb 6;15:968. doi: 10.1038/s41467-024-45067-8 (PMC10847113; doi:10.1038/s41467-024-45067-8)
Supplement: Supplementary file 3 — Description of Additional Supplementary Files [file 41467_2024_45067_MOESM3_ESM.pdf]

Description of Additional Supplementary Files

Supplementary Data Legends

Supplementary Data 1: 71-gene connectivity signature derived from SR101 xenograft scRNA-Seq data

Supplementary Data 2: 245-gene connectivity signature derived from SR101 xenograft RNA-Seq data

Supplementary Data 3: 184-gene Caprola(6) signature

Supplementary Data 4: 57-gene Caprola(on) signature

Supplementary Data 5: 171-gene calcium signature

Supplementary Data 6: Cell type signatures obtained from our patient sample snRNA-Seq data

Supplementary Data 7: RNA-Seq of CHI3L1 overexpression PDGCLs. Gene count matrix

Supplementary Data 8: 2978 DEGs in CHI3L1 overexpression PDGCLs

Supplementary Data 9: Proteomics of CHI3L1 overexpression PDGCLs. Protein intensity after normalization and imputation

Supplementary Data 10: 123 DEPs in CHI3L1 overexpression PDGCLs

Supplementary Data 11: Phospho-proteomics of CHI3L1 overexpression PDGCLs. Protein intensity after normalization and imputation

Supplementary Data 12: 152 DPPs in CHI3L1 overexpression PDGCLs

Supplementary Movie Legends

Supplementary Movie 1: Transient calcium transfer through TMs in S24 PDGCL *in vitro*.
