# Supplementary material for: A clinically applicable connectivity signature for glioblastoma includes the tumor network driver CHI3L1
Source: Nat Commun. 2024 Feb 6;15:968. doi: 10.1038/s41467-024-45067-8 (PMC10847113; doi:10.1038/s41467-024-45067-8)
Supplement: Supplementary file 6 — Reporting Summary [file 41467_2024_45067_MOESM6_ESM.pdf]

nature portfolio

Corresponding author(s): Tobias Kessler

Last updated by author(s): January 10th, 2024

Reporting Summary

Nature Portfolio wishes to improve the reproducibility of the work that we publish. This form provides structure for consistency and transparency in reporting. For further information on Nature Portfolio policies, see our Editorial Policies and the Editorial Policy Checklist.

Statistics

For all statistical analyses, confirm that the following items are present in the figure legend, table legend, main text, or Methods section.

n/a	Confirmed	
☐	☒	The exact sample size (*n*) for each experimental group/condition, given as a discrete number and unit of measurement
☐	☒	A statement on whether measurements were taken from distinct samples or whether the same sample was measured repeatedly
☐	☒	The statistical test(s) used AND whether they are one- or two-sided<br>*Only common tests should be described solely by name; describe more complex techniques in the Methods section.*
☐	☒	A description of all covariates tested
☐	☒	A description of any assumptions or corrections, such as tests of normality and adjustment for multiple comparisons
☐	☒	A full description of the statistical parameters including central tendency (e.g. means) or other basic estimates (e.g. regression coefficient) AND variation (e.g. standard deviation) or associated estimates of uncertainty (e.g. confidence intervals)
☐	☒	For null hypothesis testing, the test statistic (e.g. F, t, r) with confidence intervals, effect sizes, degrees of freedom and P value noted<br>*Give P values as exact values whenever suitable.*
☒	☐	For Bayesian analysis, information on the choice of priors and Markov chain Monte Carlo settings
☒	☐	For hierarchical and complex designs, identification of the appropriate level for tests and full reporting of outcomes
☐	☒	Estimates of effect sizes (e.g. Cohen's *d*, Pearson's *r*), indicating how they were calculated

Our web collection on statistics for biologists contains articles on many of the points above.

Software and code

Policy information about availability of computer code

Data collection	qPCR, Western Blot and Flow Cytometry<br>• QuantStudio Design & Analysis (RRID:SCR_018712, v.1.5.2, software, Thermo Fisher Scientific) for qPCR.<br>• Image Lab Touch (v.2.0.0.27, software, Bio-Rad) for Western Blots.<br>• FACSDiva (RRID:SCR_001456, v.8.0.2, software, BD Biosystems) for flow cytometry.<br>Microscopy<br>• Zen 2.6 Blue Edition® (RRID:SCR_013672, software, Zeiss) for widefield slide scans.<br>• Zeiss Zen 2012 black edition (v.8.1.0.484, software, Zeiss) for confocal and two-photon microscopy.<br>Next-generation sequencing<br>• HiSeq control (HCS, v.2.2.38 and v.3.3.52, software, Illumina) for sequencing on HiSeq2000 and HiSeq4000 as well as NovaSeq Control Software (NCS, v.1.6.0, software, Illumina) and RTA v.3.4.4 (software, Illumina) for sequencing on NovaSeq 6000.<br>• Illumina bcl2fastq pipeline for generation of the sequence data (v.2.20.0.422, Illumina).<br>• Cellranger (RRID:SCR_017344, v.1.2.0 and v.2.1.1, software, 10X Genomics) for processing scRNA-Seq and snRNA-Seq data.<br>Proteomics and Phosphoproteomics<br>• MaxQuant (RRID:SCR_014485, v.2.0.1.0, software,
Data analysis	Gene expression analysis<br>• ImageLab (v.6.0.0, software, Bio-Rad, Hercules, California, USA) for Western Blot analysis.<br>• FlowJo" (RRID:SCR_008520, v.10.8.1, software, BD Biosystems, Franklin Lakes, New Jersey, USA) for flow cytometry.<br>Microscopy<br>• Fiji (RRID:SCR_002285, v.2.0.0, software, Schindelin et al., Nat. Methods, 2012), MATLAB (RRID:SCR_001622., R2020b, software,<br>Jewwns 5upoda.a I oflop.Jod eJmeu

1
MathWorks Inc., Natick, Massachusetts, USA) and AIVIA (v.9.5, software, Leica, Wetzlar, Germany) for general image analysis using customwritten
scripts.
General omics data analysis
• R (v.3.5.1 or higher, software, https://www.R-project.org/) for gene expression analyses.
• RStudio (v.1.2.1335, software) for running R analyses.
• Python (v.3.7.11 or higher, software, https://www.python.org/) for data analyses.
• JupyterLab (v.3.3.2, software, https://jupyter.org/) for running Python analyses.
Bulk RNA-Seq
• FASTX-Toolkit (RRID:SCR_005534, software, http://hannonlab.cshl.edu/fastx_toolkit/index.html) for evaluation and control of RNASeq base quality.
• HOMER (RRID:SCR_010881, v.4.7, software, http://homer.ucsd.edu/homer/) for PolyA-tail trimming.
• STAR (RRID:SCR_004463, v.2.3, software, https://github.com/alexdobin/STAR) for mapping filtered reads against the human reference genome.
• Picard (RRID:SCR_006525, v.1.78, software, https://broadinstitute.github.io/picard/) for quality checking the mapping.
• htseq-count (RRID:SCR_011867, v.0.9.1, Python package) for generating gene count data.
• GENCODE (RRID:SCR_014966, v.19 or v.26, gene model, https://www.gencodegenes.org/) for annotation of genes.
• Subread (RRID:SCR_009803, v.1.5.3, software, https://subread.sourceforge.net/) for generating gene count data.
• DESeq2 (RRID:SCR_015687, v.1.22.2, R package) and edgeR (RRID:SCR_012802, v.3.34.1, R package) for DEG identification.
• ssGSEA (R codes from Wang Q et al., Cancer cell, 2017) for glioblastoma TCGA expression subtype assignment.
scRNA-Seq
• Cell Ranger (RRID:SCR_017344, v.2.1.1 or v.3.1.0, software, 10x Genomics, Pleasanton, California, USA) for generating gene-cell count matrix.
• scater (RRID:SCR_015954, v.1.10.1, R package) for excluding outlier cells in scRNA-Seq datasets.
• Scrublet (RRID:SCR_018098, v.0.2.1, Python package) for estimation of per-cell doublet scores and per-sample doublet score thresholds.
• Seurat (RRID:SCR_007322, v.3.1.5, R package) for additional quality control steps, identification and UMAP visualization of shared nearest neighbor unsupervised clusters, averaging of expression levels, identification of cell types and calculation of gene set scores.
• infercnv (RRID:SCR_021140, v.1.2.1, R package) for identification and validation of malignant cell clusters.
• velocyto (RRID:SCR_018167, v.0.17.15, Python package) for obtained pre-mature and mature mRNA count matrices.
• scVelo (RRID:SCR_018168, v.0.2.4, Python package) for processing the count matrices from velocyto.
• Cytoscape (RRID:SCR_OO3O32, v.3.9.0, software, https://cytoscape.org/) for visualization of PAGA graphs.
Proteomics and Phosphoproteomics
• DEP (RRID:SCR_023090, v.1.14.0, R package) for normalization of LFQ data and DEP identification.
Batch correction, accuracy prediction and statistics
• LIMMA package (RRID:SCR_010943, v.3.36.5, R package) for correction of batch effects between PDGCLxenograft models.
• caret (RRID:SCR_021138, v.6.0-80, R package) for generation of confusion matrix and prediction metrices.
• ggpubr (RRID:SCR_021139, v.0.4.0, R package) for statistical analysis.
• survival (RRID:SCR_021137, v.3.1-12, R package) and survminer (RRID:SCR_021094, v.0.4.2, R package) for patient survival analysis.
Visualization
• ggplot2 (RRID:SCR_014601, v.3.3.2, R package) for box plot and bar plot visualization.
• ComplexHeatmap (RRID:SCR_017270, v.2.5.4, R package) for Z-score scaling, centering, winsorizing and visualization of average expression levels as a heatmap.
Enrichment analysis
• clusterProfiler (RRID:SCR_016884, v.3.18.1, R package) for GO enrichment analysis.
• enrichplot (v.1.10.2, R package) for visualization of most enriched GOs.
• ShinyGO (RRID:SCR_019213, v.0.741, shiny app) for GO enrichment analysis.
• GSEA (RRID:SCR_003199, v.4.1.0, software, Broad Institute, Inc., Boston, Massachusetts, USA) for Gene Set Enrichment Analysis.

For manuscripts utilizing custom algorithms or software that are central to the research but not yet described in published literature, software must be made available to editors and reviewers. We strongly encourage code deposition in a community repository (e.g. GitHub). See the Nature Portfolio guidelines for submitting code & software for further information.

Data

Policy information about availability of data

All manuscripts must include a data availability statement. This statement should provide the following information, where applicable:

- Accession codes, unique identifiers, or web links for publicly available datasets
- A description of any restrictions on data availability
- For clinical datasets or third party data, please ensure that the statement adheres to our policy

Raw data files of all WES, bulk RNA-Seq and scRNA-Seq data generated in this study have been deposited in the European Genome-Phenome Archive database (EGA) under accession number EGAS00001007611 [https://ega-archive.org/studies/EGAS00001007611, RRID:SCR_004944]. These patients´ sensitive genetic data, including raw and processed data files, are available in line with the EGA policy and access controlled by the Data Access Committee to ensure patient privacy. All of WES, bulk RNA-Seq and scRNA-Seq data generated and analyzed in this study are provided in the Supplementary Information, Supplementary Data and Source Data. MS data generated in this study have been deposited in the ProteomeXchange Consortium (http://proteomecentral.proteomexchange.org, RRID:SCR_004055) via the PRIDE partner repository (RRID:SCR_003411) with the dataset identifier PXD044001 [https://proteomecentral.proteomexchange.org/cgi/GetDataset?ID=PXD044001]. The MS data generated in this study are provided in the Supplementary Information, Supplementary Data and Source Data.
scRNA-Seq data from the SR101 dataset can be additionally explored by an interactive webtool (https://connectivity-glioma.dkfz.de).

RNA-Seq data of the TCGA cohort was obtained from the UCSC Xena platform [https://xenabrowser.net/datapages/?cohort=GDC%20TCGA%20Lower%20Grade%20Glioma%20(LGG)&removeHub=https%3A%2F%2Fxena.treehouse.gi.ucsc.edu%3A443, https://xenabrowser.net/datapages/?cohort=GDC%20TCGA%20Glioblastoma%20(GBM)&removeHub=https%3A%2F%2Fxena.treehouse.gi.ucsc.edu%3A443, RRID:SCR_018938, https://www.cancer.gov/tcga]. RNA-Seq data of the CGGA cohort was obtained from the CGGA webpage [http://www.cgga.org.cn/download.jsp, RRID:SCR_018802]. RNA-Seq data from 31 tumor types and related

healthy tissues was obtained from the GEPIA server [http://gepia.cancer-pku.cn/detail.php?gene=chi3l1, RRID:SCR_018294]. RNA-Seq data from the GLASS cohort was obtained from the Synapse platform [https://www.synapse.org/glass, RRID:SCR_005918]. scRNA-Seq data from GBmap was obtained from the CELLxGENE data portal [https://cellxgene.cziscience.com/collections/999f2a15-3d7e-440b-96ae-2c806799c08c, RRID:SCR_021059]. GB proteogenomic cohort was obtained from the CPTAC Assay Portal [https://cptac-data-portal.georgetown.edu/cptac/s/S048] and the GDC Cancer Portal [https://portal.gdc.cancer.gov/projects/CPTAC-3, RRID:SCR_014514]. Gene sets for GSEA were obtained from gsea-msigdb.org (RRID:SCR_003199, v.4.1.0, Broad Institute). All of these datasets were accessible without any restrictions.

Research involving human participants, their data, or biological material

Policy information about studies with human participants or human data. See also policy information about sex, gender (identity/presentation), and sexual orientation and race, ethnicity and racism.

Reporting on sex and gender	As outlined in Supplementary table 4, for snRNA-Seq we collected 21 GB samples (4 females, 17 males), while for IHC we collected 6 GB samples (5 females, 1 male). No gender related issues were applied to the analysis.<br>The TCGA cohort comprised 229 GB samples (90 females, 139 males, 1 unspecified), the CCGA cohort 141 GB samples (50 females, 91 males). There was no gender-specific case filtering performed on the patients included in the GLASS, GBMap and GB proteomic cohorts, respectively. Sex and gender-related information about these patients can be found in the original publications. The female/male ratios in our datasets match previous data that reported a higher number of male patients with glioblastoma. We corrected for gender by applying a multivariate analysis in the survival analysis performed with these cohorts.
Reporting on race, ethnicity, or other socially relevant groupings	These factors were not routinely assessed for the whole cohort and therefore not considered in any of the performed analysis.
Population characteristics	For snRNA-Seq human GB samples were obtained from 20 treatment-naive patients (4 females, 16 males) and one recurrent patient with standard treatment (1 male). Median age was 61 years (range 32-80 years). For IHC human GB samples were obtained from 6 treatment-naive patients. Median age was 66 years (range 28-78 years)<br>The N2M2 pilot study included patients with MGMT promoter unmethylated tumors, leading to an enrichment of MGMT promoter unmethylated samples in our snRNA-Seq (18/21, 86%) and IHC datasets.
Recruitment	We selected specimens from GB patients enrolled in the NCT Neuro Master Match (N^2M^2) pilot study60 who have undergone surgery at the Heidelberg University Hospital and gave informed consent to exploratory molecular analyses. GB diagnosis needed to be molecularly confirmed according to the recent WHO classification and methylation profiling. Further inclusion criteria comprised the availability of matched FFT and FFPE tumor material at the Department of Neuropathology in Heidelberg, a tumor content ≥ 70% and a low percentage of necrosis of the tumor. No further criteria were applied for the case selection. The N^2M^2 pilot study included patients with MGMT promoter unmethylated tumors, leading to an enrichment of MGMT promoter unmethylated samples in our analysis (18/21, 86%). We corrected for this potential bias by validating the connectivity signature score in several other patient cohorts that did not enrich patients based on the MGMT promoter methylation status.
Ethics oversight	All patients gave informed consent either prior to inclusion to the NCT Neuro Master Match (WM') pilot study (Pfaff et al., Neuro Oncol., 2018) or to exploratory molecular analyses. The research is conducted in concordance with the declaration of Helsinki and was approved by the Ethics Committee at the University of Heidelberg, Germany (applications 206/2005 and AFmu-207/2017).

Note that full information on the approval of the study protocol must also be provided in the manuscript.

Field-specific reporting

Please select the one below that is the best fit for your research. If you are not sure, read the appropriate sections before making your selection.

☒ Life sciences ☐ Behavioural & social sciences ☐ Ecological, evolutionary & environmental sciences

For a reference copy of the document with all sections, see nature.com/documents/nr-reporting-summary-flat.pdf

Life sciences study design

All studies must disclose on these points even when the disclosure is negative.

Sample size	No statistical methods were used to predetermine sample size, but our sample sizes were selected based on those reported to generate statistically meaningful data in similar studies from our group (Osswald et al., Nature, 2015, Weil et al., Neuro Oncol., 2017, Jung et al., J Neurosci., 2017, Jung et al., Nat. Commun.,2021). Following the principles of the 3 R's, several regions were analyzed per animal allowing to obtain comparable levels of data with fewer animals ("reduction").
Data exclusions	Bulk RNA-Seq: Genes with less than 10 a total counts in all samples were discarded.<br>scRNA-Seq data: PDGCs with these uniform exclusion criteria were discarded: (1) cells with fewer than 200 or more than detected 8,000 genes. (2) cells with fewer than 500 or more than 80,000 detected counts. (3) cells with more than 10% of counts from mitochondrial genes.<br>Image analysis: To correct network parameters to be analyzed for cell density issues, ROIs with mathematical cell number outliers were excluded from further analysis. No other relevant data was excluded from analysis.
Replication	For in vitro experiments at least 2-3 independent experiments were performed with similar results. For in vivo studies at least 3 animals were

Replication	allocated per group and patient derived glioblastoma cell line (PDGCL). At least 2 independent injections were performed and concordance was found. Each human glioblastoma (GB) sample used for snRNA-Seq and IHC was considered a biological replicate as it is unique in terms of inter-patient and intra-patient tumor heterogeneity. The workflow used for nuclei isolation protocols was tested on several patient tumor samples (biological replicate) and yielded consistent results across the different samples.
Randomization	For in vitro experiments, control and intervention groups were identically seeded on the same plate and at the same time. Layout-wise, a maximally adjacent allocation of control and intervention conditions within the plate was prioritized highest and a distribution of technical replicates over the plate allowed (e.g. 1st column for control, 2nd column for intervention treatment 1, 3rd column for intervention treatment 2, 4th column for control,...). ROI positions were chosen to be relatively the same spot within each well for each condition. If there were group comparisons planned in vivo, all animals were randomly assigned into the experimental and control groups. There was no randomization of patients as all participants belonged to the same treatment group.
Blinding	In vivo orthotopic implantation of CHI3L1 Ctrl and OE PDGCs was done blinded. The setup, readout and analysis of microscopy-based in vitro and in vivo assays were also done blinded, by two independent persons and/or automated. All other wet-lab experiments were conducted with the same settings and bioinformatic analysis was performed equally with the same analysis pipeline, therefore circumventing the relevance of blinding.

Reporting for specific materials, systems and methods

We require information from authors about some types of materials, experimental systems and methods used in many studies. Here, indicate whether each material, system or method listed is relevant to your study. If you are not sure if a list item applies to your research, read the appropriate section before selecting a response.

Materials & experimental systems

n/a	Involved in the study
☐	☒ Antibodies
☐	☒ Eukaryotic cell lines
☒	☐ Palaeontology and archaeology
☐	☒ Animals and other organisms
☒	☐ Clinical data
☒	☐ Dual use research of concern
☒	☐ Plants

Methods

n/a	Involved in the study
☒	☐ ChIP-seq
☐	☒ Flow cytometry
☒	☐ MRI-based neuroimaging

Antibodies

Antibodies used	• anti-nestin antibody (#ab22035, clone 10C2, RRID:AB_446723, 1:500 dilution, Abcam)<br>• anti-CHI3L1 antibody (#ab77528, RRID: AB_2040911, 1:1250 dilution, Abcam)<br>• anti-CHI3L1 antibody (#MABC196, clone mAY, RRID:AB_2891310, 19 nM and 1:2000, Merck)<br>• IgG1 antibody (#401402, clone MG1-45, RRID:AB_2801451, 19 nM, Biolegend)<br>• anti-nestin (#ab6320, clone 196908, RRID:AB_308832, 1:500 dilution, Abcam)<br>• anti Ku-80 (#2180S, clone C48E7, RRID:AB_2218736, 1:400 dilution, Cell signaling technologies)<br>• anti-GAP43 (#8945S, clone D9C8, RRID:AB_10860076, 1:1000 dilution, Cell signaling technologies)<br>• anti-GAP43(pS41) (#ab167162, clone EPR1854(2), 1:1000 dilution, Abcam)<br>• anti-GAPDH (#97166S, clone D4C6R, RRID:AB_2756824; 1:1000 dilution, Cell signaling technologies)<br>• anti-CD31 (#AF3628, RRID:AB_2161028; 1:100 dilution, R&D Systems)<br>• anti-mouse IgG, Alexa Fluor™488-conjugated (#A11001, RRID:AB_2534069, 1:500 dilution, Thermo Fisher Scientific)<br>• anti-rabbit IgG, Alexa Fluor™546-conjugated (#A11010, RRID:AB_2534077, 1:500 dilution, Thermo Fisher Scientific)<br>• anti-mouse IgG, Alexa Fluor™546-conjugated (#A11003, RRID:AB_141370, 1:500 dilution, Thermo Fisher Scientific)<br>• anti-rabbit IgG, Alexa Fluor™633-conjugated (#A21070, RRID:AB_2535731, 1:500 dilution, Thermo Fisher Scientific)<br>• anti-mouse IgG, Alexa Fluor™633-conjugated (#A21050, RRID:AB_141431, 1:500 dilution, Thermo Fisher Scientific)<br>• anti-mouse IgG, Dy800-conjugated (#SA5-10172, RRID:AB_2556752, 1:10,000 dilution, Thermo-Fisher Scientific)<br>• anti-mouse IgG, HRP-conjugated (#NA931V, RRID:AB_772210, 1:10,000 dilution, GE Healthcare)<br>• anti-rabbit IgG, HRP-conjugated (#NA9340V, 1:10,000 dilution, GE Healthcare)
Validation	• anti-nestin antibody (#ab22035, RRID:AB_446723, Abcam)<br>Venkataramani, V. et al. Glioblastoma hijacks neuronal mechanisms for brain invasion. Cell 185, 2899-2917.e31; 10.1016/j.cell.2022.06.054 (2022)<br>• anti-CHI3L1 antibody (#ab77528, RRID: AB_2040911, Abcam)<br>Yu, J. E. et al. Anti-Chi3L1 antibody suppresses lung tumor growth and metastasis through inhibition of M2 polarization. Molecular oncology 16, 2214–2234; 10.1002/1878-0261.13152 (2022)<br>• anti-nestin (#ab6320, RRID:AB_308832, Abcam)<br>Osswald, M. et al. Brain tumour cells interconnect to a functional and resistant network. Nature 528, 93–98; 10.1038/nature16071 (2015)<br>• anti Ku-80 (#2180S, RRID:AB_2218736, Cell signaling technologies)<br>Allard, J. et al. Immunohistochemical toolkit for tracking and quantifying xenotransplanted human stem cells. Regenerative medicine 9, 437–452; 10.2217/rme.14.26 (2014)<br>• anti-CHI3L1 antibody (#MABC196, RRID:AB_2891310, Merck)<br>Morera, E. et al. YKL-40/CHI3L1 facilitates migration and invasion in HER2 overexpressing breast epithelial progenitor cells and generates a niche for capillary-like network formation. In vitro cellular & developmental biology. Animal 55, 838–853; 10.1007/

s11626-019-00403-x (2019)
Schmid, D. et al. Diagnostic biomarkers from proteomic characterization of cerebrospinal fluid in patients with brain malignancies. Journal of neurochemistry 158, 522–538; 10.1111/jnc.15350 (2021)
• anti-GAP43 (#4945S, RRID:AB_10860076, Cell signaling technologies)
Osswald, M. et al. Brain tumour cells interconnect to a functional and resistant network. Nature 528, 93–98; 10.1038/nature16071 (2015)
• anti-GAPDH (#97166S; RRID:AB_2756824; Cell signaling technologies)
Lin, K. et al. Chronic Inflammation Pathway NF-κB Cooperates with Epigenetic Reprogramming to Drive the Malignant Progression of Glioblastoma. International journal of biological sciences 18, 5770–5786; 10.7150/ijbs.73749 (2022)
• anti-CD31 (#AF3628, RRID:AB_2161028; 1:100 dilution, R&D Systems)
Jung, E. et al. Tumor cell plasticity, heterogeneity, and resistance in crucial microenvironmental niches in glioma. Nature communications 12, 1014; 10.1038/s41467-021-21117-3 (2021)
• anti-GAP43(pS41) (#ab167162, Abcam)
Manufacturer website
All seconary antibodies used in this study have been extensively tested by the respective providers to be suitable with the parameters we applied and for the respective application.

Eukaryotic cell lines

Policy information about cell lines and Sex and Gender in Research

Cell line source(s)	Patient derived glioblastoma cell lines (PDGCL) S24 and T269 were established in our laboratory (CCU Neurooncology, German Cancer Research Center, Heidelberg, Germany)<br>Lemke, D. et al. Primary glioblastoma cultures: can profiling of stem cell markers predict radiotherapy sensitivity? Journal of neurochemistry 131, 251-264; 10.1111/jnc.12802 (2014)<br>PDGCLs P3XX and BG5 were provided by H. Miletic (Department of Biomedicine, University of Bergen, Bergen, Norway)<br>Haspels, H. N., Rahman, M. A., Joseph, J. V., Gras Navarro, A. & Chekenya, M. Glioblastoma Stem-Like Cells Are More Susceptible Than Differentiated Cells to Natural Killer Cell Lysis Mediated Through Killer Immunoglobulin-Like Receptors-Human Leukocyte Antigen Ligand Mismatch and Activation Receptor-Ligand Interactions. Frontiers in immunology 9, 1345; 10.3389/fimmu.2018.01345 (2018)<br>HEK293FT were purchased (#R70007, Thermo Fisher Scientific).
Authentication	S24 (human, female), T269 (human, male), P3XX (human, male) and BG5 (human, female) were regularly checked for authenticity and absence of infections, such as mycoplasms and non-human cell contamination, as part of the multiplex cell contamination test (Multiplexion GmbH). S24, T269, P3XX and BG5 were further authenticated as GB by 850k methylation EPIC array (#WG-317-1003, Illumina) as described in Venkataramani et al., Nature, 2022 and Hausmann et al., Nature, 2022. Cell line characteristics can be found in Supplementary Table 1.
Mycoplasma contamination	The cell lines were regularly tested negative for mycoplasmen contamination.
Commonly misidentified lines (See ICLAC register)	The study did not involve commonly misidentified cell lines.

Animals and other research organisms

Policy information about studies involving animals; ARRIVE guidelines recommended for reporting animal research, and Sex and Gender in Research

Laboratory animals	Immunodeficient Crl:NMRI-Foxn1nu nude mice (RRID:MGI:5653040, male) and Crl:CD1-Foxn1nu nude mice (RRID:IMSR_CRL:086, female) were purchased from Charles River. Only mice at the age of 8 to 12 weeks were used, maintained in a specific-pathogen-free, standardized environment with 22 ±2°C temperature, 55±10% humidity, 12h light/dark cycles and fed with a standard diet according to the German Cancer Research Center guidelines.
Wild animals	The study did not involve wild animals
Reporting on sex	Immunodeficient Crl:NMRI-Foxnlnu nude mice (RRID:MGI:5653040) were male, Crl:CD1-Foxnlnu nude mice (RRID:IMSR_CRL:086) were female.
Field-collected samples	The study did not involve field-collected samples.
Ethics oversight	All in vivo experiments in this study were approved by the local authorities (Regierungsprasidium Karlsruhe, Germany) and compliant with the institutional laboratory animal research guidelines. All efforts were made to minimize animal suffering and to reduce the number of animals used according to the 3R's principles. Tumors were grown until the mice showed first symptoms or 20% weight loss were met. In none of the experiments these limits were exceeded.

Note that full information on the approval of the study protocol must also be provided in the manuscript.

Plants

Seed stocks

Report on the source of all seed stocks or other plant material used. If applicable, state the seed stock centre and catalogue number. If plant specimens were collected from the field, describe the collection location, date and sampling procedures.

Novel plant genotypes

Describe the methods by which all novel plant genotypes were produced. This includes those generated by transgenic approaches, gene editing, chemical/radiation-based mutagenesis and hybridization. For transgenic lines, describe the transformation method, the number of independent lines analyzed and the generation upon which experiments were performed. For gene-edited lines, describe the editor used, the endogenous sequence targeted for editing, the targeting guide RNA sequence (if applicable) and how the editor was applied.

Authentication

Describe any authentication procedures for each seed stock used or novel genotype generated. Describe any experiments used to assess the effect of a mutation and, where applicable, how potential secondary effects (e.g. second site T-DNA insertions, mosiacism, off-target gene editing) were examined.

Flow Cytometry

Plots

Confirm that:

☒ The axis labels state the marker and fluorochrome used (e.g. CD4-FITC).

☒ The axis scales are clearly visible. Include numbers along axes only for bottom left plot of group (a 'group' is an analysis of identical markers).

☐ All plots are contour plots with outliers or pseudocolor plots.

☐ A numerical value for number of cells or percentage (with statistics) is provided.

Methodology

Sample preparation

SR101 dye transfer in vivo model
Single cell suspensions were generated from the PDGC-tumor bearing brains utilizing a combination of gentleMACSTM Dissociator (#130-093-235, Miltenyi Biotec) and brain tumor dissociation kit (#130-095-942, Miltenyi Biotec) according to the manufacturer's recommendations. The obtained suspension was passed through 100 µm (#542000, Greiner Bio-one) and 70 µm (542070, Greiner Bio-one) strainer meshes. After subsequent centrifugation at 500 g for 5 min, the cell pellet was resuspended in FACS buffer, consisting of 1% fetal calf serum (#50615, Sigma, part of Merck) in phosphate-buffer saline (PBS). The single cell suspension was incubated with eBioscienceTM Calcein Violet 450 AM (#65-0854-39, Invitrogen, part of Thermo Fisher Scientific) and TO-PROTM-3 Iodide (#T3605, Invitrogen, part of Thermo Fisher Scientific) for 10 min on ice prior to sorting.
Caprola6 and Caprolaon PDGCs
The exact procedure is described by Huppertz et al. (reviewer only material).
Stem-like culture in vitro model of connectivity
Cells were detached using Stem-Pro Accutase" (#1110501, Thermo Fisher Scientific), washed twice with PBS and labeled with cholesterol modified oligos. Briefly, 500,000 PDGCs per line and condition were resuspended in PBS and incubated with cholesterol modified oligo solution (Integrated DNA technologies). After 3 wash cycles using 0.1% bovine serum albumin (BSA, #0163.4, Roth) in PBS and 300 g, 3 min, LleC centrifugation, DAPI was added to 100 ng/ul final concentration.
Serum-based in vitro model of connectivity
Cells were blocked with 1% BSA in PBS. PDGCs were washed with PBS and subsequently resuspended in 1.5 ml of PBS/1%BSA containing 1 µM calcein AM (#C1430, Life Technologies, part of Thermo Fisher Scientific) and 0.33 µM TO-PRO"-3 (#T3605, Invitrogen, pat of Thermo Fisher Scientific) to co-stain before sorting.

Instrument

FACSAria" cell sorter (BD Biosystems) and FACSAria" Fusion Special Order System (BD Biosystems).

Software

Data Acquisitiion: FACSDivae v.8.0.2 software (RRID:SCR_001456, BD Biosystems)
Data analysis: FlowJo" v.10.8.1 (RRID:SCR_008520, BD Biosystems)

Cell population abundance

SR101 dye transfer model
Single (SSC-Wlow), live (CalceinViolet450high, TO-PRO-3 Iodidelow), GFPhigh/SR101high: 0.1-0.6% of all events
Single (SSC-Wlow), live (CalceinViolet450high, TO-PRO-3 Iodidelow), GFPhigh/SR101low: 0.1-1.3% of all events
Caprola6 and Caprolaon PDGCs
See Huppertz et al. 2022 (reviewer only material). Briefly:
Single (SSC-Wlow), live (GFPhigh), labeling intensity (CPY-CA/GFP)high: 0.2-2.0% of all events
Single (SSC-Wlow), live (GFPhigh), labeling intensity (CPY-CA/GFP)medium: 0.5-0.4.3% of all events
Single (SSC-Wlow), live (GFPhigh), labeling intensity (CPY-CA/GFP)low: 0.2-0.7% of all events
Stem-like culture in vitro model of connectivity
Single (FSC-H/FSC-Ahigh), GFP positive (GFPhigh), live (GFPhigh, DAPIlow): 82.3-83.8% (S24), 9.7-11.4% (T269) of all events
Serum-based in vitro model of connectivity
Single (FSC-H/FSC-Ahigh), live (Calceinhigh, TO-PRO-3 Iodidelow):11.9-26.0% (S24), 0.4-11% (T269), 12.5-14.7% (P3XX), 12.6-41.2% (BG5)

Gating strategy

Describe the gating strategy used for all relevant experiments, specifying the preliminary FSC/SSC gates of the starting cell population, indicating where boundaries between "positive" and "negative" staining cell populations are defined.

☒ Tick this box to confirm that a figure exemplifying the gating strategy is provided in the Supplementary Information.
